# Assessment of First-line Therapy With Midazolam for Prehospital Seizures in Children

**DOI:** 10.1001/jamanetworkopen.2023.6990

**Published:** 2023-04-04

**Authors:** Danielle Shavit, Refael Strugo, Maya Siman-Tov, Shira Nov, Itai Shavit

**Affiliations:** 1 Department of Pediatrics, Carmel Medical Center, Haifa, Israel; 2 Magen David Adom, Tel Aviv, Israel; 3 Sackler Faculty, Public Health School, Tel-Aviv University, Tel-Aviv, Israel; 4 Division of Pediatrics, Hadassah Medical Center, Jerusalem, Israel

## Abstract

This cohort study assesses the effectiveness of midazolam treatment in terminating pediatric seizures in the prehospital setting.

## Introduction

In the prehospital setting, pediatric patients are sometimes actively seizing when emergency medical services (EMS) arrive at the scene or during transport, and if they are not effectively treated, the potential morbidity of these patients can be substantial.^1^ The National Association of State EMS Officials (NASEMSO) guideline^2^ recommends the use of intravenous midazolam in a dose of 0.1 mg/kg or intranasal or intramuscular midazolam in a dose of 0.2 mg/kg as first-line therapy for pediatric seizures. However, there are limited data on the effectiveness of this treatment. In this cohort study, we assessed the effectiveness of midazolam treatment in terminating pediatric seizures in the prehospital setting.

## Methods

This study was approved by the institutional review board of the Israel National EMS (INEMS) system and Rambam Medical Center. Informed consent was not required due to the observational and anonymous nature of the data collection. This study follows the Strengthening the Reporting of Observational Studies in Epidemiology (STROBE) reporting guidelines for cohort studies. We conducted a retrospective analysis of all patients aged 0 to 18 years for whom a mobile intensive care unit was dispatched due to a seizure between January 1, 2017, and December 31, 2019. We used the database of the INEMS system, which includes a log of dispatched calls, vehicles, paramedics’ records, and the diagnosis and treatment of all patients treated by paramedics.^3^ The primary outcome was the administration of rescue therapy, defined as an additional dose of midazolam following the first midazolam administration during the prehospital encounter.^4^ Univariable and multivariable analyses were used to identify factors associated with rescue therapy. The adjusted odds ratio (aOR) for rescue therapy was determined with a 95% CI. Data were analyzed from September 2021 to March 2022 with SPSS statistical software version 21 (SPSS-IBM).

## Results

Overall, 1172 children with a mean (SD) age of 5.7 (4.6) years, including 475 girls (40.53%) and 697 boys (59.47%), were treated with midazolam for an active seizure (Figure and Table). A total of 31 patients were treated with bag-mask ventilation, and 5 patients underwent endotracheal intubation (Figure). Median (IQR) first doses of midazolam were 0.11 (0.10-0.15) mg/kg using the intravenous route, 0.25 (0.21-0.27) mg/kg for the intranasal route, and 0.21 (0.19-0.24) mg/kg for the intramuscular route. Overall, 459 of 1172 patients (39.16%) required rescue therapy. Rescue therapy was administered to 144 of 451 patients (31.93%) using the intravenous route, 220 of 454 patients (48.46%) using the intranasal route, and 95 of 267 patients (35.58%) using the intramuscular route. Rescue therapy failed in 153 of 1172 patients (13.05%), who required a third dose of midazolam or more to terminate the seizures (Figure and Table).

**Figure.**
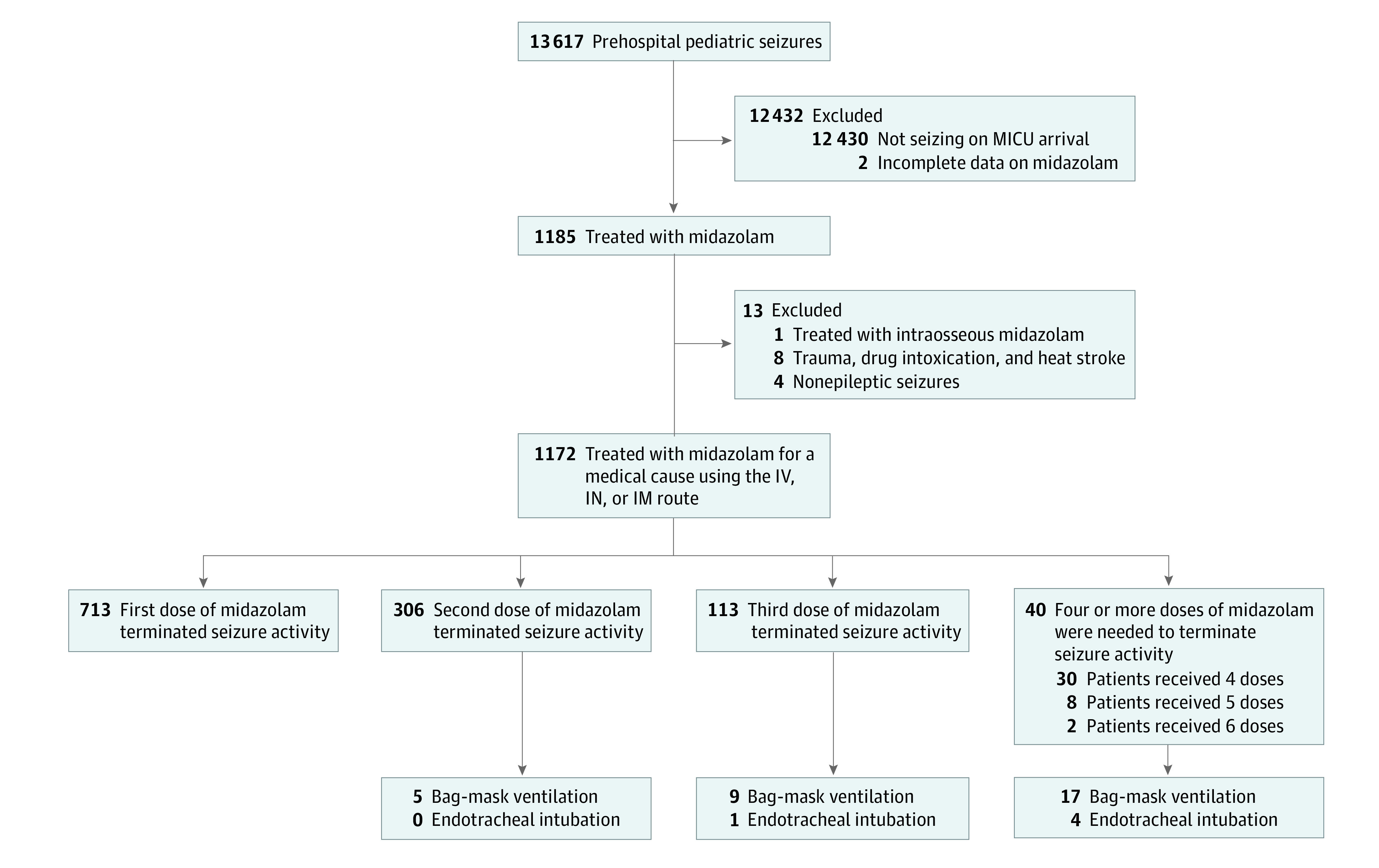
Study Flowchart IM indicates intramuscular; IN, intranasal; IV, intravenous; MICU, mobile intensive care unit.

**Table.** Baseline Characteristics of Children Treated With Midazolam for Prehospital Seizures, by Number of Doses

Characteristic	Children, No. (%) (N = 1172)			
	1 Dose (n = 713 [60.84%])	2 Doses (n = 306 [26.11%])	3 Doses (n = 113 [9.64%])	>3 Doses (n = 40 [3.41%])^a^
Age, mean (SD), y	5.9 (4.8)	5.1 (4.5)	5.4 (4.8)	5.5 (4.2)
Sex				
Male	435 (61.01)	166 (54.25)	70 (61.95)	26 (65.00)
Female	278 (38.99)	140 (45.75)	43 (38.05)	14 (35.00)
O_2_ saturation on arrival, median (IQR), %	95 (90-98)	95 (88-98)	93 (87-98)	91 (83-95)
Glucose level on arrival, median (IQR), mg/dL	122 (101-153)	140 (107-161)	142 (111-156)	143 (106-188)
MICU time of arrival from dispatch to scene, median (IQR), min	9.1 (6.1-13.4)	9.5 (6.4-14.3)	8.4 (6.2-13.1)	10.4 (6.5-14.6)
MICU time of arrival from scene to the hospital, median (IQR), min	14.3 (9.4-23.4)	14.4 (9.3-23.4)	16.3 (10.1-25.3)	17.1 (10.3-30.0)
Patient background				
Previously healthy	190 (26.65)	95 (31.05)	32 (28.32)	11 (27.50)
Convulsive disorder	259 (36.33)	109 (35.62)	51 (45.13)	18 (45.00)
Cerebral palsy	52 (7.29)	22 (7.19)	3 (2.66)	1 (2.50)
Developmental disorder	85 (11.92)	25 (8.17)	11 (9.73)	2 (5.00)
Congenital genetic or metabolic disorder	54 (7.57)	21 (6.86)	4 (3.54)	5 (12.50)
Structural brain abnormality	36 (5.05)	18 (5.88)	5 (4.43)	1 (2.50)
Other	37 (5.19)	16 (5.23)	7 (6.19)	2 (5.00)
Midazolam route of administration				
Intravenous (n = 451)	307 (43.06)	97 (31.70)	33 (29.20)	14 (35.00)
Intranasal (n = 454)	234 (32.82)	146 (47.71)	54 (47.79)	20 (50.00)
Intramuscular (n = 267)	172 (24.12)	63 (20.59)	26 (23.01)	6 (15.00)

Abbreviation: MICU, mobile intensive care unit.

SI conversion factor: To convert glucose to millimoles per liter, multiply by 0.0555.

^a^ Thirty patients received 4 doses, 8 patients received 5 doses, and 2 patients received 6 doses.

In univariable analysis, age, glucose level, route of administration, and chronic disease were significant for inclusion in the multivariable model. In multivariable analysis, only the route of administration was a significant independent factor associated with rescue therapy. Initial treatment by the intranasal and intramuscular routes had an aOR for rescue therapy of 1.65 (95% CI 1.13-2.42) and 0.97 (95% CI 0.62-1.53), respectively, compared with the intravenous route.

## Discussion

The major finding of this cohort study is the high proportion of patients (39.16%) who required rescue therapy. First-line treatment by the intranasal route was associated with a higher risk for rescue therapy, and almost half the patients (48.46%) who were initially treated by the intranasal route required rescue therapy. High proportions of patients who received first-line treatment by the intravenous (31.93%) and intramuscular route (35.58%) also required rescue therapy. Rescue therapy failed in 153 patients (13.05%). Taken together, these findings suggest that the effectiveness of midazolam to terminate prehospital pediatric seizures was suboptimal. Previous adult studies^4,5,6^ reported similar observations. A possible explanation for the inferior performance of midazolam in the prehospital setting could be the long period from the onset of seizures to the time of starting therapy. Another important finding is the substantial number of patients who needed respiratory support among those who received rescue therapy (Figure).

This study has limitations inherent to a retrospective medical record review. We did not have information on the timing of treatment regarding seizure onset and the rate of unsuccessful venous cannulation. Our results may not be generalizable to populations where home rescue treatment for seizures is common.

## Notes

Supplement.
Data Sharing Statement
