# Supplementary material for: Assessment of First-line Therapy With Midazolam for Prehospital Seizures in Children
Source: JAMA Netw Open. 2023 Apr 4;6(4):e236990. doi: 10.1001/jamanetworkopen.2023.6990 (PMC10074219; doi:10.1001/jamanetworkopen.2023.6990)
Supplement: Supplement. — Data Sharing Statement [file jamanetwopen-e236990-s001.pdf]

Data Sharing Statement

Shavit. Assessment of First-line Therapy With Midazolam for Prehospital Seizures in Children. *JAMA Netw Open*. Published April 04, 2023. doi:10.1001/jamanetworkopen.2023.6990

Data
Data available: Yes
Data types: Deidentified participant data
How to access data: The study data are available from the corresponding author (IS) upon reasonable request
When available: With publication

Supporting Documents
Document types: None

Additional Information
Who can access the data: Researchers whose proposed use of the data has been approved by the Israeli National EMS
Types of analyses: For any purpose approved by the Israeli National EMS
Mechanisms of data availability: After approval of a proposal and with the support of the principal investigator (IS)
Any additional restrictions: Any access to the data must be approved by the Israeli National EMS
